# Selective Growth of α-Sexithiophene by Using Silicon Oxides Patterns

**DOI:** 10.3390/ijms12095719

**Published:** 2011-09-06

**Authors:** Cristiano Albonetti, Marianna Barbalinardo, Silvia Milita, Massimiliano Cavallini, Fabiola Liscio, Jean-François Moulin, Fabio Biscarini

**Affiliations:** 1 CNR-ISMN, Institute for the Study of Nanostructured Materials, Via P. Gobetti 101, Bologna I-40129, Italy; E-Mails: m.barbalinardo@bo.ismn.cnr.it (M.B.); m.cavallini@bo.ismn.cnr.it (M.C.); f.biscarini@bo.ismn.cnr.it (F.B.); 2 CNR-IMM, Institute for Microelectronics and Microsystems, Via P. Gobetti 101, Bologna I-40129, Italy; E-Mails: milita@bo.imm.cnr.it (S.M.); liscio@bo.imm.cnr.it (F.L.); 3 GKSS, Forschungszentrum Geesthacht GmbH, Geesthacht D-21502, Germany; E-Mail: jean-francois.moulin@hzg.de

**Keywords:** sexithiophene, atomic force microscopy, pattern, template, annealing

## Abstract

A process for fabricating ordered organic films on large area is presented. The process allows growing sexithiophene ultra-thin films at precise locations on patterned Si/SiO*_x_* substrates by driving the orientation of growth. This process combines the parallel local anodic oxidation of Si/SiO*_x_* substrates with the selective arrangement of molecular ultra-thin film. The former is used to fabricate silicon oxide arrays of parallel lines of 400 nm in width over an area of 1 cm^2^. Selective growth arises from the interplay between kinetic growth parameters and preferential interactions with the patterned surface. The result is an ultra-thin film of organic molecules that is conformal to the features of the fabricated motives.

## 1. Introduction

New strategies for fabricating planar organic devices by placing molecules in a controlled way are the key requirements to realize electronic, optical, and magnetic devices and sensors [1]. Single molecules [2] and atoms [3] were moved, positioned and used to fabricate prototype devices for investigating the physics at the atomistic length scale. On the other hand, the electrical performances of standard organic devices [4] with micrometric size are mainly influenced by cooperative phenomena amongst molecules that occur at the nanometric/micrometric length scale. Accordingly, strategies for organizing molecules at these length scales are necessary. Unconventional lithographic techniques [5] have successfully re-organized molecular films into micrometric/nanometric structures for tuning optical [6] and electrical [7] properties, but one of the major aims of nanotechnology is to assemble devices by driving the self-organization process of molecules. The advantage of the latter approach is two-fold. On one hand, the possibility to implement additive manufacturing [8], viz. the usage of the optimum amount of material needed for the performance of the device; on the other hand, the capability of tailoring the relevant properties of those devices, such as charge mobility [9], spin coherence length [10], or radiative relaxation [11] by controlling the lateral size of the active material from nanometer to micrometer length scale. Since the lengths scales of physical phenomena in conjugated materials are in the nanometer range, optimizing the organization of the molecules at the nanometer scale would lead to an enhancement of the device’s physical properties.

Currently, few examples of fabrication approaches for conjugated materials are reported in literature [12–14]. Organic Molecular Beam Deposition (OMBD) is the most consolidated thin film growth technique for organic electronics, but it is lacking in the control of lateral size of the domains due to the coalescence of uncorrelated domains [15].

In this article we report a process for growing selectively α-sexithiophene (6T) molecules on silicon oxide patterns in order to fabricate molecularly ordered nanostructures on large superficial area (cm^2^). The method yields monolayer stripes, whose width is a few hundred nanometers. These low-dimensional structures are grown at predetermined positions on the substrate. Their shape and size are controlled by SiO*_x_* patterns fabricated on the substrate. The molecules on the pattern maintain their orientation with their long axis normal to the substrate, viz. the same orientation as those forming islands on non-patterned surface. Morphological, structural and thermal properties of 6T nanostructures were investigated by means Atomic Force Microscopy (AFM) and X-ray scattering.

## 2. Experimental and Methods

### 2.1. Fabrication of SiO_x_ Patterned Surfaces

Nanometric patterns on Si/SiO*_x_* (native) substrates were fabricated by using the parallel-local anodic oxidation (PLAO) technique [16]. This method yields silicon oxide nanostructures with height variable from few nm to 15 nm [17]. The size of the stamp (1 cm^2^) enables us to pattern the surface across centimeter length scale. To implement this technique, we built a machine to bring a metallized polydimethylsiloxane (PDMS) stamp in contact with the silicon surface. The PDMS stamps (Sylgard 184 Down Corning) were prepared by replica molding [18] using, as a master, the surface of a blank compact disk (CD). On the centimeter length scale, the CD is composed of an array of parallel stripes with spatial periodicity (λ) of 1.4 μm, full width at half maximum (FWHM) of 400 nm and height (*h*) of 200 nm.

**Figure 1:**
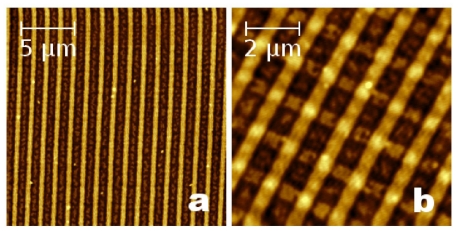
Si oxide patterns on SiO*_x_* surfaces fabricated by using parallel-local anodic oxidation (PLAO) technique. (**a**) Array of Si oxide stripes with λ = 1.28 μm, full width at half maximum (FWHM) ≈ 400 nm and *h* = (74 ± 4) Å; and (**b**) Si oxide grid (holes size ≈ 650 nm, bar width ≈ 500 nm) fabricated by turning of 90° the Si/SiO*_x_* substrate.

The machine, with prearranged stamp and substrate, was inserted in a sealed chamber with controlled relative humidity. The stamp and the sample were gently placed in contact by means of the downward motion of a micrometric screw. The oxide nanostructures (stripes—Figure 1a) were fabricated when a bias voltage of 36 V is applied between the stamp and the silicon substrate for 2 min, with a relative humidity of 90%. The flexibility of the stamp enables a homogeneous conformal contact with the silicon surface, resulting in an excellent reproducibility of the process. In addition, this allowed us to fabricate a complex nanostructure (grid—Figure 1b) by means of two subsequent oxidations with the same stamp (just rotating the sample of 90°).

### 2.2. Organic Molecular Beam Deposition of 6T Ultra-Thin Films

Ultra-thin films of 6T molecules (Sigma-Aldrich) were grown by OMBD in high vacuum (base pressure 2 × 10^−8^ mbar) [19]. The film thickness *t* ranges between 10 Å (surface coverage Θ ≈ 40%, *i.e.*, sub-monolayer thick film) and 200 Å (≈8 monolayer (ML)) [20]. Substrates used were Si (*n*^++^-type, doping > 5·× 10^19^ cm^−3^, *ρ* ≤ 0.0015 Ω·cm) with native SiO*_x_* either patterned (P) or non-patterned (NP), held at 120 °C during the molecular deposition [21]. The deposition rate *r* was kept constant at a value ≈0.04 Å/s. Both *t* and *r* were measured *in-situ* with a quartz crystal micro balance (QCM) and cross-checked with Atomic Force Microscopy (AFM) measurements.

**Figure 2:**
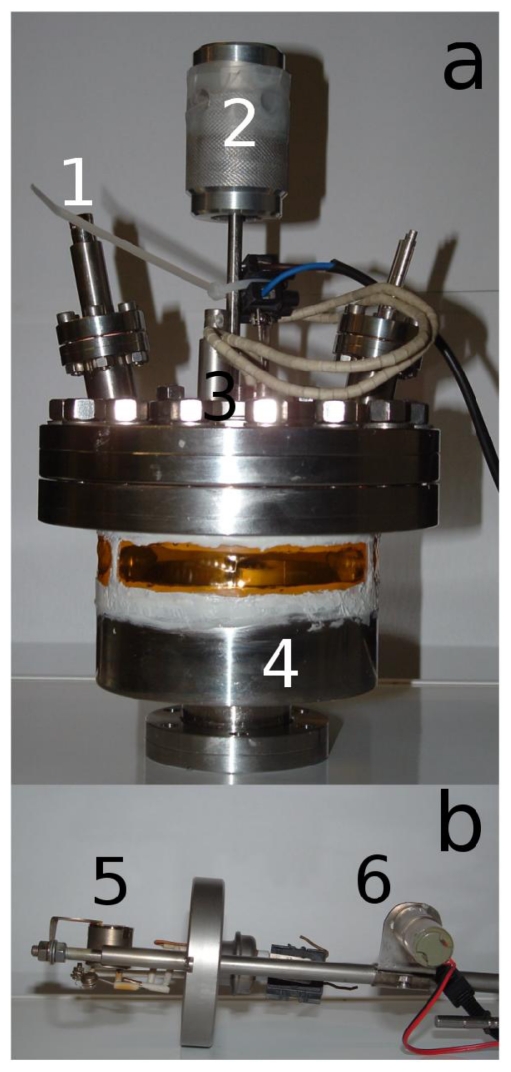
(**a**) Upper part of the Organic Molecular Beam Deposition (OMBD) system with custom-tailored flange (CF100) consisting of sample heater (3), shutter (2) and quartz crystal micro balance (QCM) (1) with water cooling circuit (on the right). The flange is mounted on a custom-tailored reduction from CF100 to CF40 (4) with three kapton windows because kapton is X-ray transparent; and (**b**) Special designed Knudsen cell (5) with motorized shutter (6). The complete OMBD system is compactly furnished, just 50 cm high and 25 cm wide.

A custom OMBD system was home-built to perform *in-situ* and in real-time X-ray measurements on growing 6T films (Figure 2a). 6T molecules were sublimed under high vacuum (base pressure 2 × 10^−6^ mbar) by using a specially designed Knudsen cell (Figure 2b).

### 2.3. Atomic Force Microscopy Measurements

The AFM employed is a Smena model (NT-MDT, Moscow, Russia) working in Intermittent Contact Mode (ICM) [22]. Cantilevers for ICM were silicon cantilevers (NSG11-NT-MDT, two cantilevers for chip) with elastic constant *k* = 12(6) N/m and a resonance frequency ω_0_ = 255(150) kHz.

The topography of 6T films was investigated by AFM under ambient conditions, as well as their thermal stability [23]. The sample was directly attached to the top of a heating stage (maximum temperature *T*_max_ = 200 °C) via silver paint (SPI supplies). The substrate temperature *T*_sub_ was raised stepwise from RT to 120 °C (Δ*T* steps of 10 °C) and held one hour in order to reach the thermal equilibrium of the cantilever-sample system. 6T film was topographically characterized in ICM for increasing *T*_sub_ (RT, 30 °C, 40 °C…).

### 2.4. Image Processing of 6T Islands: Angular Orientation, Roughness, Relative Position and Surface Coverage

**Figure 3:**
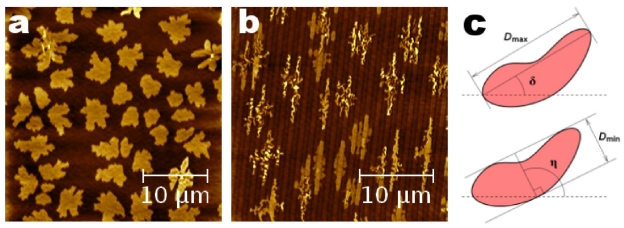
6T sub-monolayer film (≈0.4 ML) grown on Si/SiO*_x_* surface where the half of the surface was patterned. AFM images of 6T islands on NP (**a**) and P (**b**) surfaces. (**c**) Maximum and minimum bounding dimensions and angles of a grain (courteously from reference [27]).

6T molecules deposited on chemically inert, NP, SiO*_x_* surface, firstly form stable nucleus of molecules that evolve as islands during the deposition (Figure 3a) and lastly coalesce one each other to form a continuous organic film [24,25]. Conversely, molecules deposited on P SiO*_x_* surface form elongated islands in the stripes direction where molecules are mainly grew on top of the SiO*_x_* stripes (Figure 3b).

6T islands grown on both P and NP SiO*_x_* surfaces were imaged by AFM and morphologically analyzed by measuring: (a) angular orientations with respect to a preferential direction (morphological effect induced by stripes); (b) roughness and relative position induced by the *T*_sub_ changes; and (c) Θ evolution. AFM images were quantitatively analyzed by using Gwyddion image software (version 2.25) [26].

The angular orientations were measured by using the Grain Analysis toolbox of Gwyddion [27]. Firstly, 6T islands were indentified by means of a height threshold algorithm. Regardless of the island’s shape, maximum (*D*_max_) and minimum (*D*_min_) bounding dimensions were defined and associated to the longest dimension and the maximum width (orthogonal to *D*_max_) of 6T islands (Figure 3c. Accordingly, two angles (δ and η = 90° + δ) were defined with respect to the horizontal direction (dashed lines in Figure 3c).

AFM images were digitally rotated for matching the direction of the stripes to the horizontal one and δ (in degrees—°) was measured as the angle between this direction and *D*_max_. Data were plotted in polar graph where the *x*-axis runs from −90° to 90° (0° corresponds to a perfect alignment between island and stripe) and the *y*-axis represents the ordinal number of islands. The degree of the island’s alignment was quantified through the Order Parameter (S), as calculated from the equation [28]:

(1) $$S=\frac{3}{2}<{\text{cos}}^{2}\;\delta >-\frac{1}{2}$$

where *S* is the mean value of the second Legendre Polynomial (dimensionless). In particular, *S* = 1 corresponds to a perfect alignment of 6T islands along the stripes, while if *S* goes to 0 islands have an isotropic distribution on the surface.

The Root Mean Square Roughness *R*_q_ of both SiO*_x_* surface and top surfaces of islands was measured by Gwyddion. It is defined as the average of the measured height deviations taken within the evaluation length and measured from the mean line.

The relative spatial position of each island was measured by using the software ImageJ (version 1.44p) [29]. The geometric centre of a 6T crystal (C) was taken as a reference point due to its geometrical stability on *T*_sub_ changes. Straight lines were drawn to connect C with the geometric centers of the surrounding 6T islands and their lengths were measured by ImageJ. Such analysis was performed on AFM images attained at RT, 50, 70, 80, 90, 110 and 120 °C.

The surface coverage Θ was calculated by using the height threshold algorithm to identify the 6T islands. For definition, Θ is the ratio between the sum of the islands areas projected on the SiO*_x_* surface and the total area of the image.

### 2.5. *In-Situ* and Real-Time X-Ray Measurements

X-ray scattering measurements *in-situ* and in real-time were performed during the deposition of 6T molecules on P (stripes) and NP Si/SiO*_x_* substrates (1 cm^2^). X-ray experiments were performed at the ID32 beam line of the European Synchrotron Radiation Facility (ESRF—Grenoble, France) by using the OMBD system (cf. 2.2) mounted on a horizontal Huber 6-circle diffractometer [30].

A Si(111) double crystal monochromator was used to select the 0.6667 Ǻ wavelength. A set of vertical (*v*) and horizontal (*h*) slits were used to define the beam size (*v* = 400 μm, *h* = 20 μm). Such small *h* reduces the X-ray damages of the organic film [31] (each organic film spectrum is recorded from a surface slice 20 μm wide) and allows us to record several spectra on not-irradiated surface regions just by shifting horizontally the sample. Diffraction patterns both in specular out-of-plane and in Grazing Incidence Diffraction (GID) geometries have been recorded by means of a NaI scintillator point detector.

## 3. Results and Discussion

### 3.1. Pattern *vs.* Film Thickness: from Sub-Monolayer to Bulk

The selective growth of 6T molecules was investigated with respect to the nominal deposited thickness *t* (expressed in Å and ML), as measured by the QCM. To study the nucleation stage, firstly we limit our investigations to the early stage of growth (*t* = 10, Å ≈ 0.35 ML), *i.e.*, at low coverage Θ ≤ 40%, when neither coalescence nor Ostwald ripening of the islands are observed [32]. As shown in Figure 3, 6T molecules were deposited on a tailored substrate with oxide stripes on the half of the SiO*_x_* surface. In the P part (Figure 3b), they nucleate and grew selectively on the top surface of the stripes, resulting in stretched islands compared with the rounded ones grown on the NP part (Figure 3a). The selective growth arises from the interplay between kinetic growth parameters and preferential interactions with the patterned surface [12].

On P surface, the surface coverage Θ is reduced (Θ_NP_ ≈ 40%, Θ_P_ ≈ 20%) as well as the number of islands *N* (from *N*_NP_ ≈ 50 to *N*_P_ ≈ 30). In either case, the height of the islands is constant to (40 ± 4) Å, so an enhanced molecular diffusion induced by the pattern should be excluded. As reported elsewhere [33], this height value exceeds largely the length of the 6T molecule measured by means X-ray [30] (24 Å), indicating the presence of a second molecular layer on the top of the 6T islands [34]. Accordingly, Θ and *N* differences might be ascribed either to the geometry/morphology of the SiO*_x_* pattern or the molecular growth mechanism.

In the matter of the geometrical shape of the SiO*_x_* pattern, it is composed by an array of stripes with λ ≈ 1.4 μm, FWHM ≈ 500 nm, *h* = (20 ± 6) Å, top width ≈460 nm (top surface of the stripes) and basal width ≈650 nm (interstitial surface between stripes). The oblique angle between top and basal vertexes is ≈0.3°, which is too small for assigning the observed Θ difference to an image artefact [35].

Concerning the morphology of the SiO*_x_* pattern, the roughness *R_q_* on both top and basal surfaces was ≈3 Å and ≈12 Å, respectively [36]. As reported elsewhere [37], the molecular diffusion is favoured on low roughness surfaces (top surface of the stripes) with respect to high roughness surface (basal surface). On the basis of this simple morphological argument, the stretched shape of 6T islands can be explained, but neither geometrical nor morphological aspects of the SiO*_x_* pattern are responsible for the Θ and *N* decreasing.

On the other hand, the molecular growth mechanism depends on both surface chemical composition [19] and growth parameters (*r* [32] and *T*_sub_ [21]). In our experiments, *r* and *T*_sub_ were kept constant (cf. 2.2). As shown by both Spectrophotometer and X-ray Photoemission Spectroscopy (XPS) measurements [16], the oxide fabricated by PLAO has an average thickness of ≈130 Å (the pristine oxide has ≈20 Å), exhibits the 30% of porosity more than the thermal one and it is mainly composed by Si dioxide (SiO_2_) with some small contributions to other Si chemical states. Its water contact angle (WCA) [38] is ≈100° while the one measured on the native Si oxide is ≈60° (for Si specification cf. 2.2). The increase of the WCA from native to anodized oxide reflects a surface chemical composition change. Accordingly, the islands density *N* (and consequently Θ) is reduced because of the lower sticking coefficient [19,39].

For increasing film thickness, the selective effect of the pattern was investigated by X-ray scattering and AFM. X-ray scattering measurements *in-situ* and in real-time were performed on NP and P (stripes with λ = 1.4 μm, FWHM ≈ 500 nm and *h* = (22 ± 2) Å) Si/SiO*_x_* substrates (1 cm^2^). The sample shutter was moved laterally (6 steps of about 35 mm) to screen partially the P surface to the molecular beam (film thickness sequence: 13, 33, 45, 52, 81 and 108 Ǻ), thus the film thickness increases gradually from 13 Ǻ (≈0.5 ML—thinner area) to 108 Ǻ (≈4.5 ML—thickest area). The shutter of the Knudsen cell was closed when additional X-ray measurements were required.

**Figure 4:**
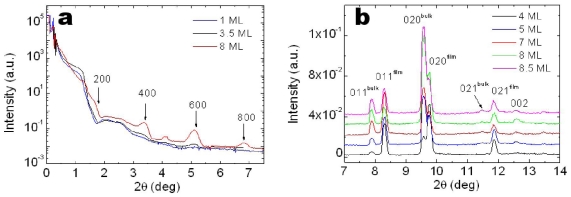
Series of XRD patterns collected in (**a**) specular and (**b**) GID geometries of 6T thin films deposited on NP Si/SiO*_x_* with thickness ranging from 1 to 8.5 MLs. The GID were performed with α_i_ = 0.1°.

Firstly, 6T films deposited on NP SiO*_x_* substrate have been investigated as a reference. Figure 4a shows specular X-ray diffraction patterns recorded for thin films (*t* = 212 Ǻ ≈ 8.5 ML). Weak Bragg peaks were recorded for 3.5 ML thick film, which became quite pronounced for film 8 ML thick. The presence of the only (h00) reflections and their angular positions showed a monoclinic unit cell for 6T molecules like the one in the so called Low Temperature phase [40] (*a* = 44.708 Ǻ, *b* = 7.851 Ǻ, *c* = 6.029 Ǻ, β = 90.76°). Along the direction orthogonal to the surface, molecules stack preferentially along their long axis, almost perpendicular to the substrate. The film consists of crystallites whose vertical domain size is of the same order of the film thickness (8.5 ML thick films), as estimated by the FWHM of the diffraction peak [41]. Additional information has been obtained analyzing the GID patterns performed with an incident angle α_i_ = 0.1°, just above the critical angle of the silicon substrate (Figure 4b).

The presence of (0kl) reflections confirms the crystallite preferential orientation with the (bc) plane parallel to the substrate surface. The peaks arising from thin film and bulk phases coexist for all the film thicknesses. For thinner film, the film phase largely dominates while it progressively decreases to the bulk one for increasing thickness.

Combining out-of plane and in-plane geometries for X-ray scattering we have investigated the growth of 6T molecules on P SiO*_x_* surface (*t* = 108, Å ≈4.5 ML). The 6T film shows the same crystal structure, preferential orientation and co-presence of film and bulk phases, as determined for the film deposited on NP SiO*_x_* substrate. In order to verify a possible orientation with respect to the SiO*_x_* stripes, azimuthal scans have been performed in GID geometry, *i.e.*, scattering intensity has been acquired by keeping the detector at the Bragg angle of the (011) and (020) reflections and turning the sample around the direction orthogonal to the surface [42]. The intensity invariance along these scans indicates no preferential orientation with respect to the stripes, differently from what observed on 6T film grown on SiO_2_ grooves (λ = 400 nm, width 200 nm and depth 100 Å) where the packing of molecules shows graphoepitaxy [43] with the *b* axis of the cell parallel to the groove direction.

**Figure 5:**
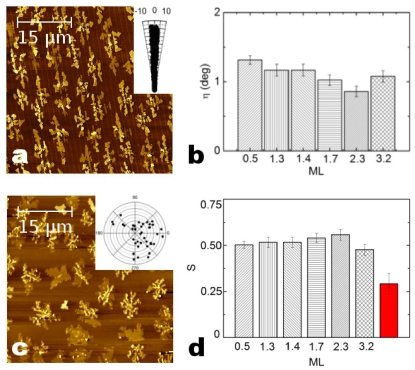
AFM images of 6T films 31 Å thick grown on P surface (**a**) and 13 Å thick grown on NP surface (**c**). The islands alignment along stripes was evaluated by means of both the angle η (**b**) and the order parameter (**d**). Inset figure shows η for each imaged 6T islands for P (**a**) and NP (**c**) surfaces.

Conversely to X-ray measurements, *ex-situ* AFM measurements on the 6T film grew on the P surface show a selective growth of the film up to ≈3.2 ML, thickness for which the pattern effect disappears (Figure 5a). For film thicknesses ranging from 13 Å ≈ 0.5 ML to 81 Å ≈ 3.2 ML, 6T islands were aligned along the stripes, moving away from the stripes direction of η ≈ 1° (polar plot of Figure 5a,b). On the contrary, islands grown on a NP surface region showed a random spatial distribution (Figure 5c and its polar plot). The order parameter S is constant to ≈0.5 for all thicknesses (Figure 5d) while it decreases about the 50% (≈0.25) for 6T islands grown on the NP surface (Figure 5d—depicted bar not-labeled in the X-axis). As described above, 6T islands nucleate and grew preferentially on the top surface of the stripes (that cover ≈50% of the total surface area) and their coverage Θ_stripe_ increases in the early stage of the growth (*t* = 13 Å ≈ 0.5 ML → Θ_stripe_ = 50%, 33 Å ≈ 1.4 ML → Θ_stripe_ = 63%) till the soil of 45 Å ≈ 1.9 ML where Θ_stripe_ is 100%.

### 3.2. Thermal Annealing and Thermal Stability of 6T Films

For every 6T film thicknesses, *ex-situ* AFM images show 6T crystals grew on the surface (Figure 5a). Their average lateral size increases (from 1 to 5 μm^2^) with the film thickness (from 13 Å ≈ 0.5 ML to 81 Å ≈ 3.2 ML). As reported in literature [44], the growth of 6T crystals is due to the thermal annealing of the film *during* the growth process. In particular, the annealing process promotes a morphological transition of the film from grains to *lamellae* (highly crystalline aggregates). In our case, the time spent for X-ray measurements *during* the growth (13 h) is equivalent to an annealing process of the 6T film at 120 °C. Moreover, the selective growth induced by the SiO*_x_* pattern addresses further a *lamellae* growth [21].

**Figure 6:**
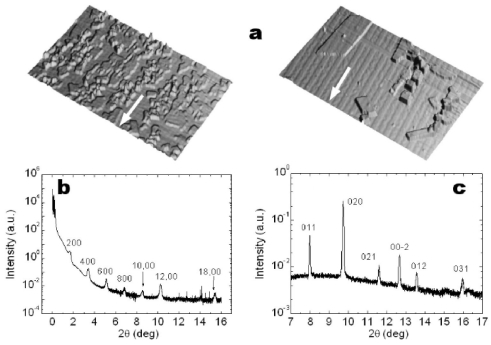
(**a**) 6T crystallites on P SiO*_x_* surfaces (arrows show the stripes direction). 3D topographic images of a 6T film 52 Å thick and deposited at *r* ≈ 0.04 Å/s (Left) and a 6T film 61 Å thick and deposited at ≈0.007 Å/s (Right). Specular (**b**) and GID (**c**) diffraction patterns recorded for 6T deposited on P SiO*_x_* surface at *r* ≈ 0.007 Å/s.

6T films with *lamellae* morphology can be obtained by either reducing the deposition rate [45] or increasing the substrate temperature up to 150 °C [46]. Accordingly, the *lamellae* growth was investigated for a 6T film deposited on identical P SiO*_x_* surface held to 120 °C. The film thickness (61 Å ≈ 2.5 ML) was comparable to the previous ones but grown with lower both deposition rate (*r* = 0.007 Å/s) and thermal annealing time (3 h). The annealing/pattern effect was successfully reproduced and 6T crystals with lateral up to 20 μm^2^ were grown on the patterned surface (Figure 6a). The crystallites are aligned along the stripes direction and show strong anisotropy in some preferential directions. As measured from AFM images, the polar plot shows 4 preferential angles (30°, 60°, 90° and 120°) with respect to the stripes direction, accordingly to some preferential directions of growth ([010], [110], [100] and [−1,1,0]) [46]. Those values are in agreement with previous result obtained on 6T crystals grown on mica [46].

6T crystals were investigated by *ex-situ* X-ray measurements. Molecules are arranged in the bulk phase structure and they have very large lateral and vertical domain sizes, respectively D_//_ = 800 Å and D_⊥_ = 483 Ǻ (as estimated from the FWHM of the 011 and 200 peaks reported in Figure 6b,c).

The thermal stability of the 6T film *after* the growth process was investigated by AFM performed in air and for increasing *T*_sub_ (RT, 50, 70, 80, 90, 110 and 120 °C). A sub-ML thick film (8 Å) grown on P SiO*_x_* surface (stripes with λ ≈ 1.6 μm, FWHM ≈ 580 nm, *h* = (13 ± 2) Å) was chosen to stress as much as possible the morphological transformations of the film induced by the temperature.

As reported in literature, in air the 6T polycrystalline powder melt at 305 °C [47,48], well above our operative conditions (120 °C at most). Moreover, in the temperature range RT–120, polymeric thin films (<100 nm) have shown de-wetting phenomena [49] induced by thermal fluctuations [50].

**Figure 7:**
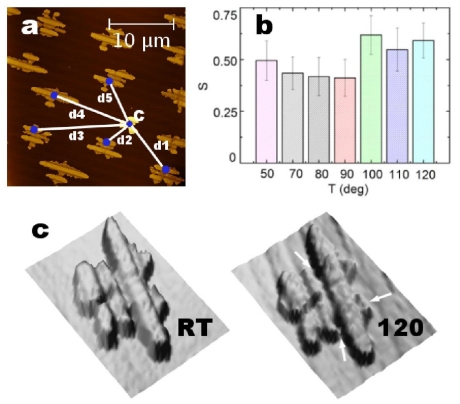
(**a**) Topographic image of 6T film recorded at RT. Islands relative distances *d_n_* (with *n* = 1, …5) are sketched. (**b**) Order parameter *vs. T*_sub_ (**c**) 3D topographic images of an island recorded at RT (left) and 120 °C (right). The top surface of 6T island at 120 °C shows a clear roughening induced by SD.

The thermal stability of 6T film was firstly investigated by measuring the relative distances *d_n_* among 6T islands (where n is the ordinal number of islands). Each *d_n_* is the distance between the geometric centre of an island and the 6T crystal (Figure 7a). Regardless *T*_sub_, *d_n_* are constant, viz. the geometrical positions of the islands on the P SiO*_x_* surface are fixed.

In order to reach the thermal equilibrium (cf. 2.3), topographic images of 6T film were recorded with the substrate kept to *T*_sub_ for one hour. As a consequence, the film was subjected to a long post-annealing process in air (9 h: 6 temperatures–6 h, plus 6 AFM images–3 h) which is, *de facto*, a thermal annealing. Usually, such post-processes improve the crystallographic order and change the morphological properties of the film [51]. The effect of this post-process can be evaluated by measuring the fractal dimension *D*_f_ that, in the case of sub-monolayer organic films [52], provides information on the growth process [53]. 6T islands have *D*_f-NP_ = (1.310 ± 0.006) and *D*_f-P_ = (1.77 ± 0.03) for NP and P surfaces, respectively and these values are constant with respect to *T*_sub_. Nevertheless, *D*_f_ suggests a morphological transition induced by either pattern and surface chemical composition (cf. 3.1): the growth mechanism evolves from a molecular diffusion at island edges [54] (formation of non-fractal structures) towards a diffusion limited aggregation scenario [55] (formation of fractal structures).

In spite of the invariance of *d_n_* and *D*_f_ to *T*_sub_, the order parameter S changes abruptly between 90 and 100 °C. As shown in Figure 7b, S is ≈0.46 up to 90 °C (in agreement with the previous value observed for increasing thickness) then it changes to ≈0.56 in the temperature range 90–120 °C. In addition, AFM images show a roughness increasing of the islands top surface from ≈2.8 Å to ≈5.2 Å (Figure 7c) [36]. The latter observation suggests the spinodal dewetting (SD) of the 6T film. In SD, height variation patterns were created due to the film undulations induced by *T*_sub_ [56]. In our case, SD was activated above 90 °C and 6T molecules were reorganized towards the stripes (arrows in Figure 7c). As a consequence, on one hand, the top surface roughness of 6T islands increases while, on the other hand, the molecules moved from the basal surface caused S increases.

### 3.3. Enhancement of the 6T Selective Growth by Means of Electric Field and Grid Pattern

Electrostatic interactions between a charged substrate surface and organic molecules are used for controlled placement of nanoscale building blocks [57]. This was done by creating charge patterns, *i.e.*, electrostatic templates, on the substrate surface and letting the organic molecules interact with the charge patterns.

In SiO*_x_* patterns fabricated by local anodic oxidation, the existence of space charges has been proved [58], whereupon both topographical and electrical effects are responsible for the 6T arrangement on the pattern. In particular, the space charge influence is two-fold: on one hand, it enhances the placement of 6T molecules while on the other hand it competes with the molecular diffusion driven by the substrate temperature. This is true especially where the space charge shows some non-homogeneity [59].

The latter problem should be solved to increase the amount of 6T molecules placed on the pattern. For this reason, P SiO*_x_* substrates were investigated by Phase-Electrostatic Force Microscopy (Phase-EFM) [60]. The tip was grounded while the P substrate was biased. A series of EFM images (not shown here) are recorded for increasing bias, from 0 to 20 V. The electrostatic contrast on top of the SiO*_x_* pattern shows an increased charge homogeneity, which saturates at 15 V.

**Figure 8:**
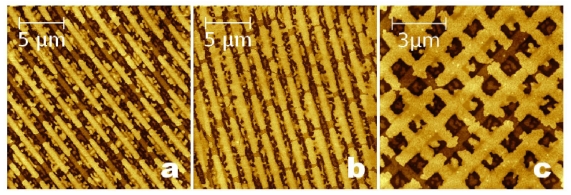
Topographic images of 6T film on P SiO*_x_* surface (**a**) grounded and (**b**) biased; (**c**) complete molecular arrangement on SiO*_x_* grid.

In order to investigate the enhanced effect driven by the electrical potential, 6T film 12 Å thick (Θ ≈ 50%) was grown on two identical P SiO*_x_* surfaces (stripes with λ ≈ 1.4 μm, FWHM ≈ 550 nm, *h* = (22 ± 1) Å), one grounded and the other one 15V biased. The surface coverage Θ was chosen just above the low coverage limit (Θ = 40 %) for reaching the islands coalescence through the electric field. As shown in Figure 8a,b, the surface coverage is enhanced because molecules are mostly placed on top of the stripes and they are covered (Θ_stripe_) for about the 90 % of their surface (Table 1).

As observed in Figure 8a, 6T film (12 Å thick) grown on grounded surface shows stretched islands in which the part of the island residing on top of the stripes ranges from 1 to 5 μm. This range of lengths suggests another way to drive topographically the molecular arrangement using a pattern with comparable (or smaller) features. An almost complete arrangement of 6T molecules was reached by using a grid with holes size ≈650 nm and bar width ≈500 nm (Figure 8c).

## 4. Conclusions

We have demonstrated a process for the fabrication of ordered low-dimensional structures of 6T molecules on large area. The process is based on the integration of SiO*_x_* substrates patterns fabricated by using the parallel local anodic oxidation technique, and the selective growth of molecular thin films. The main result is the control of 6T islands anisotropy by topographic patterns; in such a way that we have successfully self-assembled both stripes and networks of 6T islands. Such morphological control is thickness-dependent and, on SiO*_x_* stripes, it reaches 100% of covered stripes for ≈1.9 ML thick film. For thicker film, the pattern effect disappears. The SiO*_x_* pattern does not induce any 6T crystalline deformation or changes in molecular orientation. Indeed, the crystalline structure presents the same features observed on 6T thin film grown on non-patterned substrate. Patterned 6T films are thermally stable and the best patterns coverage with a lower amount of deposited materials has been reached by enhancing the pattern effect with the electric field and different geometries (grid). Because molecules stand with their long axes almost orthogonal to the substrate, the present process is suitable to fabricate substrate with precise topographical and chemical patterns. Moreover, parallel arrays (or networks) of nanometer-size 6T stripes placed between two planar electrodes are suitable for systematic studies of charge transport in conjugated organic molecular wires.

## Figures and Tables

**Table 1:** Angle η between *D*_max_ (cf. 2.4) and the stripes direction, order parameter *S* and stripes surface coverage Θ_P_ for grounded and biased samples.

	η (°)	*S*	Θ_stripe_ (%)
Ground	0.81 ± 0.08	0.62 ± 0.03	73 ± 5
15 V	0.6 ± 0.1	0.75 ± 0.03	86 ± 4
